# The Crimino-Legal Responsibility of the Mentally Defective Psychologically Considered

**Published:** 1898-08-27

**Authors:** A. R. Douglas

**Affiliations:** Deputy Medical Officer H.M. Prison, Portland, late Assistant Medical Officer East Riding Asylum and Royal Albert Asylum, Lancaster


### Medical Progress and Hospital Clinics.

[*The Editor will be glad to receive offers of co-operation and contributions from members of the profession. All letters should be addressed to* The Editor, at the Office, 28 & 29, Southampton Street, Strand, London, W.C.]

### THE CRIMINO-LEGAL RESPONSIBILITY OF THE MENTALLY DEFECTIVE PSYCHOLOGICALLY CONSIDERED.

By A. R. Douglas, Deputy Medical Officer H.M. Prison, Portland, late Assistant Medical Officer East Riding Asylum and Royal Albert Asylum, Lancaster.

In this paper I do not propose to dwell upon the idiot or the imbecile under care in an asylum. There are a large number of individuals of a lesser degree of intellectual defect of whose existence little or nothing is generally known, and for whom there is at present no legal recognition whatever. They are quite as numerous as those who are certifiable as imbecile or insane, and are in my opinion just as deserving of consideration. Many years ago the legal and social irresponsibility of the lunatic was made clear, and due regard was secured for his condition in the criminal courts. Later came an era which brought about an amelioration in the condition of the idiot and the imbecile. Asylums were built and endowed for their reception, which are really schools for the education and training of the improveable cases, and the results of the efforts made in this direction have in every instance been most encouraging. It is to be regretted, however, that those unfortunate beings who are not certifiable as imbecile or insane have not been participators in equal ratio in the beneficent alteration which humanity and civilisation have brought about in the condition of the lunatic and the imbecile. At the present time the gaol and the convict prison is the final destination of many of these cases—and why? They are not criminals in the strict sense of the term; they become gaol-birds and convicts because their congenitally feeble mental organisation renders them incapable of a normal conception of the moral and social laws. Uncontrolled by the inhibitory faculties which govern the processes of ideation and thought evolution in the healthy brain, their abnormal mental action runs rampant and unchecked in distorted channels, and they find their outlets in the commission of acts which society recognises as criminal. Judges hold that such individuals are responsible persons and fit to plead; they receive their sentence, probably one of penal servitude, and after observation in prison their defect gradually becomes apparent.

In this paper it is not my intention to describe the characteristics which prevail amongst these mentally defective offenders, as I have already done this elsewhere; but one is considerably aided in appreciating and understanding their position by an inquiry, as far as this is possible, into their family history. In a large proportion of instances it will be found that many have sprung from an insane or alcoholic stock, and in several a criminal heredity has been noted. In nearly all there is a marked development of sexual nisus, and it is not unreasonable to suppose that such individuals procreate descendants analogous to themselves. The punitive and preventive element embodied in their sentences has literally no meaning for them whatever. Few will deny their guilt, and equally few will express or evince either shame or regret. I have a vivid recollection of a man who worked out a long sentence for an indecent assault. He was a very typical example of his class, and upon one occasion he conversed freely with me upon the subject of his crime. He admitted that he was guilty, did not evince the slightest degree of modesty, but appeared to delight in details of a salacious character. He regarded his imprisonment as a very great wrong, and certainly did not convey the impression that he considered the serious offence of which he was guilty as anything to be particularly ashamed of. Now this man was fairly normal in appearance, and there was but little in his manner and conversation to lead one to regard him upon cursory examination as mentally defective, but after a longer acquaintance with him one gradually began to notice his defects. It is this very lack of palpable indications of mental weakness which is common to all such cases that operates so powerfully in causing them to be regarded as *compos mentis* and responsible for their acts. There is no doubt that certain so-called "criminals by passion" conform to this type, and it is in this connection that I shall briefly allude to the case of Prince, who murdered Mr. Terriss, and whom I have frequently heard spoken of as being "mentally defective." This man was a lunatic, and bore no direct analogy to the group of cases which I am at present endeavouring to describe. At the trial it was proved that the prisoner had been peculiar and strange since his childhood, that he had been subject to violent ebullitions of temper, and that he had given expression to delusions. The testimony of his relatives and friends pointed to the supposition that he must have been on the borderland of insanity for some years, and that he was actually insane for some time before he murdered Mr. Terriss. How this man had not years ago found his way to the asylum is, I confess, remarkable.

But to return to the consideration of the mentally-defective offender, if he is to be regarded as a criminal, under what division are we going to place him? It is surely impossible to relegate him to either the instinctive or habitual classes of criminals, and it would be absurd to attempt to put such a case in the same category as that generally accepted type of the instinctive criminal—Thomas Wainewright, poet, critic, and poisoner; or with that notorious example of the habitual criminal, Charles Peace, the burglar and murderer. It would be equally impossible to classify our weak-minded offender with the "occasional criminal." How, then, are we going to describe him? In my opinion, he is not a criminal at all, but an individual who is prone to offences by reason of his being the victim of his own degraded individuality consequent upon a defectively balanced mind.

Finally, what remedies can be suggested which would accord some kind of recognition for these cases? The question is a very difficult one indeed, and there are so many obstacles to be overcome before one can expect to see any line of action taken which would be of material and practical utility. In the first place, something must be done to ensure a definite form of nomenclature which would accurately describe their mental condition as distinguished from that of the lunatic or imbecile. The term "weak-mindedness," with certain others, is at present much too elastic and comprehensive in its variety of acceptation to render it of any value in point of exactness. I think that a step in the right direction would be to subject such persons to a more extended period of mental observation and of a more searching nature before being brought up for trial. Here might be urged the apparent impracticability of such a course, inasmuch as the appearance, manner, and conversation of such offenders afford but little indication of mental deficiency! But I think that the application of such a system would not present difficulties which would prove insurmountable, and it would be worthy of trial. Again, the success which has attended the formation of the epileptic colony at Chalfont St. Peter might well encourage the establishment of a similar one for the cases we are now considering. It would soon become self-supporting, and amongst other advantages it would have the important effect of checking in some degree the wholesale propagation of a new generation of faulty and defective brains, and here might be drafted those cases which evaded detection before coming to prison, instead of their being discharged only to return time after time.

Lastly, with respect to the great educational establishments for the education and training of imbeciles, from these institutions many a lad of practically normal appearance, but of pronounced mental defect, enters the world, so to speak, after his period of training is completed. He is undoubtedly improved thereby, but it is absurd to suppose that lads of this type, crippled with their mental defect, can compete in the struggle for existence with people of normal brains. A large number of them are failures away from the discipline and control of an institution, and ultimately undoubtedly furnish their quota of mentally-defective "criminals."

